# Incomplete Concordance Between Nominal Eosinophilic Labels and Molecular Burden in Chronic Rhinosinusitis with Nasal Polyps

**DOI:** 10.3390/biomedicines14061189

**Published:** 2026-05-25

**Authors:** Shiwang Tan, Ju Lai, Heng Zhi, Wei Tang, Ling Jin, Shaoqing Yu

**Affiliations:** 1 Department of Otolaryngology, Head & Neck Surgery, Tongji Hospital, School of Medicine, Tongji University, Shanghai 200065, China; 2 Department of Allergy, Tongji Hospital, School of Medicine, Tongji University, Shanghai 200065, China

**Keywords:** chronic rhinosinusitis with nasal polyps, eosinophilic subclassification, molecular burden, single-cell RNA sequencing, digital spatial profiling, epithelial remodeling

## Abstract

**Background/Objectives**: Chronic rhinosinusitis with nasal polyps (CRSwNP) is a heterogeneous inflammatory disease in which eosinophilic subclassification is widely used for clinical stratification. However, it remains unclear how closely nominal histologic eosinophilic labels reflect the broader molecular organization of diseased tissue. **Methods**: We performed an inference-based integrative analysis of public datasets spanning discovery single-cell RNA sequencing (scRNA-seq), independent scRNA-seq validation, GeoMx digital spatial profiling, and bulk transcriptomic replication cohorts. A sample-level molecular burden framework was constructed using four dimensions: type 2 inflammation, epithelial injury/remodeling, extracellular-matrix remodeling, and barrier/defense impairment. Composite burden and component-level features were then examined across nominal eosinophilic categories, epithelial states, spatial compartments, and independent bulk cohorts. **Results**: Nominal eosinophilic labels were directionally informative but incompletely concordant with molecular burden. In the discovery cohort, eosinophilic CRSwNP samples were enriched toward the higher-burden end, whereas nominally non-eosinophilic CRSwNP samples extended across the intermediate-to-high burden range. Across discovery and validation scRNA-seq datasets, GeoMx spatial profiling, and independent bulk cohorts, the most reproducible burden-associated signals centered on epithelial injury/remodeling-like programs and related remodeling features. In the epithelial compartment, higher burden was associated with epithelial state reorganization, stronger wounding-associated activity, and trajectory-linked glandular/secretory remodeling. Independent validation and spatial analyses further supported epithelial wounding-, barrier-, and myeloid remodeling-related features, whereas type 2 context signals were directionally consistent but less uniform across platforms. In bulk replication, composite burden, epithelial wounding, and myeloid remodeling were more consistent across cohorts than type 2 context alone. **Conclusions**: Nominal eosinophilic labels in CRSwNP capture clinically relevant but incomplete information about underlying tissue biology. Epithelial injury/remodeling-like programs and remodeling-linked myeloid features emerged as the most stable organizational axes of molecular burden across public multimodal datasets. These findings support a graded, multidimensional view of CRSwNP and may complement, rather than replace, conventional pathology-based eosinophilic subclassification.

## 1. Introduction

Chronic rhinosinusitis with nasal polyps (CRSwNP) is a heterogeneous inflammatory disease of the upper airway. It typically presents with nasal obstruction, rhinorrhea, and olfactory dysfunction, and it can markedly impair health-related quality of life (HRQoL) [1,2,3]. Although current treatment includes intranasal and systemic corticosteroids, endoscopic sinus surgery, and, more recently, biologics targeting type 2 inflammation, many patients with severe disease still relapse and require repeated systemic treatment or revision surgery [1,4,5,6]. These ongoing clinical challenges reflect the biological heterogeneity of CRSwNP, which involves immune endotypes, epithelial dysfunction, and tissue remodeling [1,7,8].

Eosinophilic subclassification remains one of the most widely used ways to stratify CRSwNP. In many studies, eosinophilic disease is associated with stronger type 2 immune signals, a higher risk of recurrence, and possible differences in treatment response [9,10,11,12]. However, eosinophilic classification is not standardized across studies or regions. The JESREC framework, for example, combines clinical and radiologic features with peripheral blood eosinophilia to identify eosinophilic CRS [10], whereas other approaches rely more directly on tissue eosinophil proportion or eosinophil counts per high-power field, with variable cutoffs that may also relate to recurrence risk [11,12,13]. The updated Chinese CRS guideline also notes the lack of a unified standard and summarizes several commonly used definitions [9]. Comparative studies further suggest that different tissue-eosinophilia thresholds identify overlapping, but not identical, clinical and immunologic states. Taken together, eosinophilic labels remain clinically useful, but they are only imperfectly standardized surrogates of the underlying tissue biology [12,13].

CRSwNP pathology is unlikely to be explained by eosinophil burden alone. Growing evidence points to epithelial barrier impairment, abnormal epithelial repair, and stromal remodeling as key features of the disease that go beyond traditional histologic classification [14,15,16]. Single-cell, spatial, and multi-scale transcriptomic studies also support this view, showing heterogeneous immune–epithelial interactions and remodeling programs across tissue states. These findings suggest that nominal histologic eosinophilic labels capture only part of the molecular heterogeneity of CRSwNP, including epithelial dysfunction, remodeling, and immune–epithelial organization [8,16,17]. We therefore asked how closely nominal histologic eosinophilic labels correspond to a broader multi-axis molecular burden framework across public single-cell, spatial, and bulk CRSwNP datasets, and whether molecular stratification could provide information beyond conventional pathology-based subclassification. We hypothesized that eosinophilic labels would still be informative, but incomplete, meaning that molecular burden would vary in a graded manner and align only partly with nominal eosinophilic categories across cohorts and platforms [8].

## 2. Materials and Methods

### 2.1. Study Design

This study integrated public transcriptomic datasets encompassing discovery single-cell RNA sequencing (scRNA-seq), independent scRNA-seq validation, GeoMx digital spatial profiling, and bulk transcriptomic replication cohorts. The aim was to determine how closely nominal histologic eosinophilic labels correspond to a broader multi-axis molecular burden framework across datasets and platforms.

### 2.2. Public Datasets and Overall Analytical Framework

The study comprised four analytical layers. First, we used a discovery scRNA-seq cohort [18] to construct the formal four-axis molecular burden framework and define core epithelial states and transcriptional programs. Second, we analyzed an independent validation scRNA-seq cohort as a conceptually aligned external support layer for selected epithelial and immune features. Third, a GeoMx digital spatial profiling dataset [16] provided orthogonal compartment-resolved support using platform-adapted modules. Finally, independent bulk transcriptomic cohorts served as a framework-level replication layer to evaluate incomplete concordance between nominal eosinophilic labels and molecular burden in both label-explicit cohorts, including GSE72713 [19] and Ishino [20], and control-versus-polyp datasets.

### 2.3. Construction of the Molecular Burden Framework

The formal four-axis molecular burden framework was constructed only in the discovery scRNA-seq cohort (n = 21 samples: Control, 5; CRSsNP, 5; non-eosinophilic CRSwNP, 5; eosinophilic CRSwNP, 6), where sample-level pseudobulk profiles allowed all prespecified dimensions to be evaluated in the same analytical layer. The framework was designed as an exploratory transcriptomic representation of recurrent CRSwNP pathobiology, not as an exhaustive classification system or a clinically validated score. We selected four dimensions based on prior evidence that CRSwNP tissue organization reflects inflammatory context, epithelial injury and repair, stromal/extracellular-matrix remodeling, and epithelial barrier or host-defense dysfunction.

Gene sets were obtained with “msigdbr” from MSigDB. Type 2 inflammatory context was represented by “GOBP_TYPE_2_IMMUNE_RESPONSE”; epithelial injury/remodeling by “HALLMARK_EPITHELIAL_MESENCHYMAL_TRANSITION”; extracellular-matrix remodeling by “REACTOME_EXTRACELLULAR_MATRIX_ORGANIZATION”; and epithelial barrier/defense by “HALLMARK_APICAL_JUNCTION”, “HALLMARK_APICAL_SURFACE”, and “GOBP_DEFENSE_RESPONSE_TO_BACTERIUM”. Gene-set identifiers and the number of genes retained after filtering to the discovery pseudobulk matrix are provided in Supplementary Table S1.

Single-cell counts were aggregated by sample to generate pseudobulk expression matrices. Pseudobulk counts were normalized using trimmed mean of M-values (TMM) normalization and transformed to log counts per million. Signature scores were calculated with GSVA using the ssGSEA method and were z-scaled across samples. Barrier integrity and antibacterial defense were treated as protective programs; therefore, their signs were inverted and averaged to generate a burden-oriented barrier/defense impairment score. The discovery composite molecular burden was then defined as the unweighted mean of the four standardized burden-oriented dimensions: type 2 inflammation, epithelial injury/remodeling, extracellular-matrix remodeling, and barrier/defense impairment. Equal weighting was chosen as a parsimonious analytical choice to avoid assigning unsupported biological weights to individual axes. Samples were ranked by this composite score and, when needed for visualization, grouped into tertiles. These tertiles were operational strata for displaying graded molecular organization and were not intended to define fixed biological thresholds.

### 2.4. Consistency and Sensitivity Analyses

We evaluated the discovery burden representation using several descriptive consistency and sensitivity analyses. First, we calculated component-to-composite and inter-component correlations to assess internal consistency and overlap among the four prespecified dimensions. Second, we performed leave-one-component-out (LOCO) analyses, recalculating the composite burden after sequential removal of each component. Because LOCO correlations are partly expected when standardized correlated components are averaged, these analyses were interpreted as internal consistency checks rather than independent proof of robustness.

To directly test whether the burden ordering depended on specific weighting choices, we performed alternative burden formulation analyses. These included an epithelial injury/remodeling downweighted composite, a type 2 inflammation downweighted composite, and a rank-based composite using the average rank across the four burden-oriented components. We compared each alternative formulation with the original discovery composite using Pearson and Spearman correlations and assessed the overlap of the high-burden tertile. We also examined median-based burden grouping and alternative rank-based stratification as additional checks of the discovery burden pattern.

### 2.5. Discovery Epithelial Single-Cell Analysis

To identify cellular correlates of disease burden, we extracted epithelial cells from the discovery cohort and analyzed them as a dedicated Seurat object. We then mapped sample-level burden assignments back to individual cells using matched identifiers. Discovery epithelial-state annotation was guided by canonical marker expression together with prior nasal/CRS single-cell epithelial atlases defining related basal, secretory, goblet, glandular, and ciliated compartments and their disease-associated remodeling states [21]. State composition was quantified as the cellular proportion of each state within each sample.

We evaluated functional epithelial programs using the AddModuleScore function in Seurat [22]. Unlike the inverted scaling used for the composite framework, we preserved these module scores in their native direction to accurately reflect underlying transcriptional activity. Scores were averaged across epithelial cells at the sample level prior to group comparisons. For trajectory inference using Slingshot [23], basal cells were used as the root based on their progenitor-like identity in prior CRS epithelial single-cell atlases and established airway epithelial lineage organization. Glandular and ciliated states were treated as terminal branches, and glandular-branch marker dynamics were examined as supportive evidence for epithelial state organization while recognizing that inferred trajectory orientation is conditional on root designation. To provide tissue-level qualitative support for these epithelial remodeling-associated findings, we examined SERPINB4 immunofluorescence in relation to KRT5-defined basal/progenitor-associated epithelial compartments. Immunofluorescence staining was performed using anti-KRT5 antibody (Abcam, Cambridge, UK; ab52635; rabbit monoclonal; clone EP1601Y; 1:100) and anti-SERPINB4 antibody (OriGene Technologies, Rockville, MD, USA; UM500016; mouse monoclonal; clone UMAB16; 1:100). Nuclei were counterstained with DAPI (1 μg/mL; Thermo Fisher Scientific, Waltham, MA, USA; D1306), and sections were mounted with antifade mounting medium (Beyotime Biotechnology, Shanghai, China; P0126). Images were acquired using an Olympus BX53 fluorescence microscope (Olympus Corporation, Tokyo, Japan) and analyzed using ImageJ software (version 1.54g; National Institutes of Health, Bethesda, MD, USA; https://imagej.nih.gov/ij/). In this qualitative panel, SERPINB4 was interpreted as a remodeling/pathology-associated epithelial signal, with KRT5 serving as a contextual basal/progenitor anchor rather than as a stand-alone classification system.

### 2.6. Independent Validation scRNA-Seq Analysis

The independent validation scRNA-seq cohort was used as a conceptually aligned external support layer rather than as a de novo reconstruction of the four-axis discovery composite. Exact transfer of the discovery framework was not feasible because the validation dataset did not provide explicit eosinophilic subclassifications and did not contain the same full set of compartments needed to reconstruct all four discovery axes, including a fibroblast/stromal compartment comparable to the extracellular-matrix component in the discovery framework. We therefore retained the source study’s tissue and disease annotations and mapped them to burden-oriented comparison groups to test whether the main epithelial and immune features identified in the discovery analysis showed compatible behavior in an external dataset.

Within the epithelial compartment, we calculated module scores for wounding, barrier-associated, and secretory/remodeling-associated programs, together with basal, ciliated, and secretory-state modules. Wounding and barrier-associated programs were derived from public gene sets where possible, whereas state-associated modules used curated marker panels suitable for the validation epithelial atlas. Scores were summarized at the sample level before statistical comparison. Within immune cells, analyses focused on a curated myeloid remodeling panel and a curated type 2 T/NK context panel. These validation analyses were interpreted as external support for selected framework-related features rather than exact replication of the discovery composite burden score.

### 2.7. GeoMx Spatial Profiling Analysis

The GeoMx digital spatial profiling dataset was analyzed as an orthogonal compartment-resolved support layer based on multiplex digital spatial profiling methodology [24]. Because the GeoMx dataset used predefined epithelial, immune, and macrophage-enriched compartments and did not provide a fibroblast/stromal compartment comparable to the discovery extracellular-matrix axis, the four-axis discovery composite could not be transferred directly. Instead, we evaluated platform-adapted, conceptually aligned modules within their corresponding spatial compartments: epithelial injury/remodeling and epithelial barrier programs in epithelial regions, macrophage remodeling in macrophage-enriched regions, and type 2 immune context in immune regions. Region of interest (ROI)-level summaries were used as the analytical units. Cross-compartment relationships were assessed descriptively to determine whether epithelial injury/remodeling features showed partial coupling with macrophage remodeling or type 2 immune context across ROIs.

### 2.8. Bulk Transcriptomic Analysis

Bulk transcriptomic cohorts were used as a framework-level replication layer using platform-adapted signatures. Two label-explicit cohorts, GSE72713 (n = 9; Control, 3; non-eosinophilic CRSwNP, 3; eosinophilic CRSwNP, 3) and Ishino (n = 26; source groups Control, 6; nonECRS, 8; ECRS/Asp, 12, treated here as nominally non-eosinophilic and eosinophilic CRSwNP labels), were used to examine whether nominal non-eosinophilic and eosinophilic CRSwNP labels showed incomplete concordance with bulk molecular burden. Additional control-versus-polyp cohorts included GSE179265 (CT, 7; NP, 17), GSE136825 (CT, 28; NP, 42), and GSE36830 (CT, 6; NP, 12) to assess broader case-versus-control generalizability. Because bulk RNA-seq averages multiple cell types and does not provide compartment-resolved fibroblast, epithelial, myeloid, or T/NK states, these analyses were not treated as an exact reconstruction of the discovery scRNA-seq four-axis composite.

The main bulk analysis used four conceptually aligned signatures. ‘Type2_Context’ was a curated type 2 context panel (‘IL4’, ‘IL5’, ‘IL13’, ‘HPGDS’, ‘GATA3’). ‘Myeloid_Remodeling’ was a curated myeloid/remodeling panel (‘CD68’, ‘CCL18’, ‘MMP9’, ‘FN1’, ‘SPP1’). ‘Wounding_Program’ was based on the public ‘GOBP_RESPONSE_TO_WOUNDING’ gene set, and ‘Barrier_Associated_Program’ was based on ‘HALLMARK_APICAL_JUNCTION’. Signature scores were calculated by ssGSEA and z-scaled within each cohort. A platform-adapted bulk composite was calculated as the sum of scaled type 2 context, wounding, and myeloid remodeling scores minus the scaled barrier-associated score. This bulk composite was used to evaluate directionality and relative effect magnitude rather than to claim exact identity with the discovery scRNA-seq composite.

To assess whether bulk conclusions depended on selected curated panels, we performed alternative-signature sensitivity analyses. These alternative definitions were used only for sensitivity analyses and did not replace the main bulk analysis. The type 2 context panel was replaced by a public type 2 immune response gene set, and the myeloid panel was expanded to include additional myeloid/remodeling markers (‘CD163’, ‘MRC1’, ‘MSR1’, ‘POSTN’) in addition to the main myeloid genes. We then recalculated alternative bulk composites and compared original and alternative scores using Spearman correlations, effect sizes, and high-burden tertile overlap.

### 2.9. Statistical Analysis

The sample or ROI, rather than individual cells, was used as the primary analytical unit for burden and module comparisons. In the discovery framework, scRNA-seq data were aggregated to sample-level pseudobulk profiles before GSVA/ssGSEA scoring. In epithelial, immune, and validation scRNA-seq analyses, cell-level module scores were summarized at the sample level before group comparisons. In GeoMx analyses, ROI-level or compartment-resolved ROI summaries were used. This strategy was used to reduce pseudoreplication arising from treating cells or subregions from the same biological sample as independent observations.

Pairwise group comparisons were performed primarily using two-sided Wilcoxon rank-sum tests. P values were adjusted for multiple testing using the Benjamini–Hochberg false discovery rate method where applicable. Effect sizes were summarized using Cohen’s d for case-versus-control or high-versus-low comparisons. For the bulk multi-cohort effect-size summary, 95% confidence intervals for Cohen’s d were estimated by bootstrap resampling. Correlations were assessed using Pearson or Spearman correlation as appropriate. Spatial cross-compartment correlations were interpreted descriptively. Analyses and visualization were performed in R (version 4.2.2; R Foundation for Statistical Computing, Vienna, Austria; https://www.r-project.org/) using Seurat (version 4.4.0; https://satijalab.org/seurat/), GSVA (version 1.46.0), edgeR (version 3.40.2), msigdbr (version 25.1.1), ggplot2 (version 4.0.2), ggpubr (version 0.6.3), dplyr (version 1.2.0), tidyr (version 1.3.2), Slingshot (version 2.6.0), and related packages.

## 3. Results

### 3.1. Public Transcriptomic Data Reveal a Graded and Partially Overlapping Molecular Burden Landscape Across Histologic Labels in the Discovery scRNA-Seq Cohort

**Figure 1:**
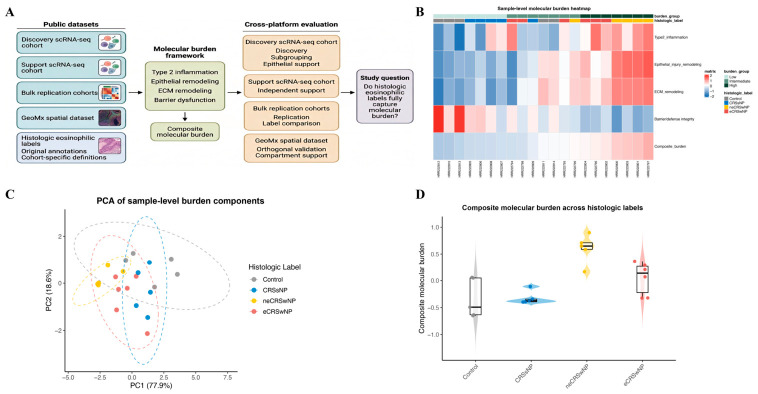
Public transcriptomic analysis reveals a graded molecular burden landscape across histologic labels in the discovery scRNA-seq cohort. (**A**) Study overview. The formal four-axis molecular burden framework was constructed in the discovery scRNA-seq cohort using sample-level pseudobulk profiles and predefined gene-set-based signatures representing type 2 inflammation, epithelial injury/remodeling, extracellular-matrix remodeling, and barrier/defense impairment. External validation scRNA-seq, GeoMx spatial profiling, and bulk transcriptomic datasets were analyzed as conceptually aligned support layers rather than exact reconstructions of the discovery composite. (**B**) Sample-level heatmap of discovery burden components and composite molecular burden. Samples were ordered by composite burden. The barrier-related row is displayed as protective barrier/defense integrity, whereas the composite framework uses its inverse burden-oriented representation. (**C**) Principal component analysis of discovery sample-level burden components. Samples showed a graded and partially overlapping distribution across histologic labels rather than complete categorical separation. (**D**) Composite molecular burden across histologic labels in the discovery cohort.

To examine how histologic eosinophilic labels relate to transcriptomic burden in CRSwNP, we built a sample-level molecular burden framework in the discovery scRNA-seq cohort using four curated dimensions: type 2 inflammatory context, epithelial injury/remodeling, extracellular matrix remodeling, and barrier/defense impairment (Figure 1A). Because eosinophilic subclassification is clinically useful but not uniformly defined across studies, we then asked how closely these labels matched transcriptomic burden within this dataset (Figure S1A).

Burden components showed a graded pattern across samples rather than a dichotomous split. Control samples were mostly located at the lower-burden end, whereas CRSwNP samples spread across a wider molecular range. Although histologically eosinophilic CRSwNP samples were enriched toward the higher-burden end, they were not completely separated from nominally non-eosinophilic CRSwNP, leaving substantial overlap between the two groups (Figure 1B).

Principal component analysis revealed a similar pattern. Control and CRSsNP samples clustered toward the lower-burden region, whereas neCRSwNP and eCRSwNP samples were distributed more broadly and exhibited partial overlap (Figure 1C). Composite molecular burden also increased across disease groups, but overlap remained between nominally non-eosinophilic and eosinophilic CRSwNP (Figure 1D). Correlation analyses supported the overall framework, confirming the expected relationships between individual components and the composite score, while also suggesting some overlap among selected dimensions (Figure S1B,C). These results indicate that histologic eosinophilic labels were related to molecular burden in the discovery cohort, but the correspondence was incomplete.

### 3.2. Nominally Non-Eosinophilic CRSwNP Is Represented Across the Intermediate-to-High Molecular Burden Range in the Discovery Cohort

**Figure 2:**
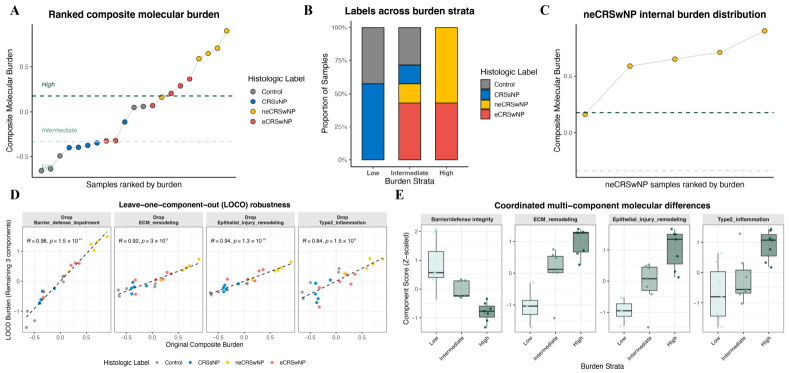
Nominally non-eosinophilic CRSwNP is represented across the intermediate-to-high molecular burden range in the discovery cohort. (**A**) Discovery cohort samples were ranked according to the composite molecular burden score. Dashed lines indicate the tertile-based cutoffs used to define low-, intermediate-, and high-burden strata. (**B**) Distribution of histologic labels across the burden strata defined in panel (**A**). Control and CRSsNP samples predominated in the low-burden stratum, whereas nominally non-eosinophilic and eosinophilic CRSwNP samples were concentrated in the intermediate- and high-burden strata. (**C**) Ranked distribution of composite molecular burden within nominally non-eosinophilic CRSwNP samples, illustrating within-group heterogeneity across the intermediate-to-high burden range. Horizontal dashed lines mark the same tertile-based cutoffs as in panel (**A**). (**D**) Leave-one-component-out analysis of the composite burden framework. Concordance between the original composite burden score and leave-one-component-out scores is shown as an internal consistency check for the burden-based representation. (**E**) Component-wise quantitative comparison across burden strata. Type 2 inflammation, epithelial injury/remodeling, and extracellular-matrix remodeling increased toward the higher-burden end, whereas the barrier/defense integrity component showed a contrasting directional pattern consistent with reduced protective function across burden strata.

We then investigated this discrepancy directly by ranking discovery cohort samples according to composite molecular burden. Although eosinophilic CRSwNP samples were more frequently located toward the higher-burden end, nominally non-eosinophilic CRSwNP samples were not restricted to the lower-burden range (Figure 2A). Using tertile-based grouping, we divided samples into low-, intermediate-, and high-burden strata. Control and CRSsNP samples predominantly occupied the low-burden stratum, whereas nominally non-eosinophilic and eosinophilic CRSwNP samples were concentrated in the intermediate- and high-burden strata (Figure 2B). This pattern remained consistent when we used a median-based alternative grouping scheme (Figure S2B). When nominally non-eosinophilic CRSwNP samples were examined separately, their composite scores still spanned a broad range within the intermediate-to-high group, indicating substantial within-group heterogeneity (Figure 2C).

To evaluate whether the discovery burden ordering depended strongly on the equal-weighted composite, we first examined component-to-composite relationships and LOCO scores. The LOCO scores remained correlated with the original composite after each component was removed (Figure 2D), but we interpret this result cautiously because high concordance is partly expected when correlated standardized variables are averaged. We therefore added alternative burden formulations to test weighting sensitivity more directly. Downweighting the epithelial injury/remodeling component, downweighting the type 2 component, and using a rank-based composite all preserved the overall sample ordering relative to the original composite (Spearman r = 0.896–0.983; Pearson r = 0.965–0.992). The overlap with the original high-burden tertile ranged from 0.714 to 0.857 across these alternative formulations (Figure S2D). Component-wise burden-stratum comparisons are summarized in Figure 2E, and additional component-level, rank-based, and alternative-formulation views are provided in Figure S2A,C,D. These results indicate that the discovery burden landscape was not driven solely by one reasonable weighting choice, while also reinforcing that the composite should be interpreted as an exploratory sample-ordering framework rather than a fixed clinical score.

### 3.3. Single-Cell Analysis Links Higher Molecular Burden to Epithelial State Reorganization, Injury-Associated Programs, and Trajectory-Associated Remodeling

**Figure 3:**
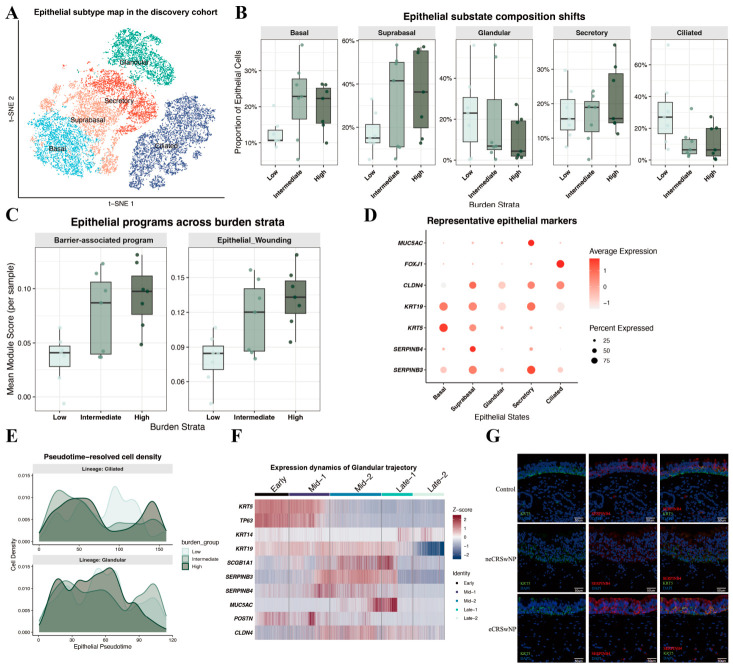
Single-cell analysis links higher molecular burden to epithelial state reorganization, injury-associated programs, and trajectory-associated remodeling. (**A**) Epithelial-focused t-SNE of the discovery scRNA-seq cohort. Epithelial cells were extracted from the overall atlas and re-analyzed to define the major epithelial substates shown in the projection. (**B**) Epithelial substate composition across burden groups. Higher molecular burden was associated with redistribution of epithelial substates rather than a uniform epithelial landscape across samples. (**C**) Epithelial wounding and barrier-associated programs across burden groups. Epithelial cells from higher-burden samples showed stronger wounding-associated transcriptional programs together with altered barrier-associated epithelial programs. (**D**) Representative epithelial markers associated with epithelial state annotation and burden-linked epithelial features. Marker-level expression patterns supported both epithelial substate annotation and the program-level differences highlighted in panel (**C**). (**E**) Pseudotime-resolved epithelial cell density across representative epithelial lineages. Burden groups showed distinct distributions along epithelial pseudotime, providing trajectory-associated support for epithelial state reorganization. (**F**) Gene-expression dynamics along the glandular epithelial trajectory. Ordered expression changes in basal, transitional, and secretory/pathology-associated markers provided branch-level support for burden-associated glandular/secretory remodeling. (**G**) Representative qualitative tissue-level immunofluorescence support for epithelial remodeling-associated findings in CRSwNP. Representative fields of control mucosa, non-eosinophilic CRSwNP, and eosinophilic CRSwNP tissues showing SERPINB4 immunofluorescence in relation to KRT5-defined basal/progenitor-associated epithelial compartments are shown. SERPINB4 is interpreted here as a remodeling/pathology-associated epithelial marker. Images are presented as qualitative visual support for the epithelial remodeling axis and do not carry quantitative inferential weight. Scale bars, 50 μm.

To examine the cellular basis of the molecular burden framework, we focused on the epithelial compartment in the discovery scRNA-seq cohort. Epithelial cells were isolated from the full atlas and subclustered to identify the major epithelial substates in the dataset (Figure 3A and Figure S3A). Canonical markers supporting these annotations are shown in Figure 3D and Figure S3B. Compared with lower-burden groups, higher-burden samples showed an altered epithelial state distribution, characterized by a relative depletion of ciliated cells and an expansion of suprabasal and secretory states (Figure 3B). These findings indicate that a higher burden was associated with epithelial state reorganization rather than a simple shift in overall epithelial composition.

At the program level, epithelial cells from higher-burden samples exhibited stronger wounding transcriptional activity alongside altered barrier programs (Figure 3C). Representative marker expression patterns confirmed these features across distinct substates (Figure 3D). At the sample level, epithelial wounding also correlated positively with composite molecular burden (Figure S3D). We then examined whether these epithelial differences extended to trajectory organization. Pseudotime analysis revealed distinct burden-group distributions along representative epithelial lineages (Figure 3E; global lineage structure in Figure S3C). Given the prominent remodeling features within the glandular branch, we further examined its gene-expression dynamics. The ordered expression of basal, transitional, and secretory pathology markers, coupled with burden-stratified smoothed trajectories, substantiated this glandular/secretory remodeling (Figure 3F and Figure S3E). Collectively, higher molecular burden in the epithelial compartment was characterized mainly by state redistribution, amplified injury programs, and glandular/secretory remodeling.

To provide qualitative tissue-level support for the epithelial remodeling-associated findings, we examined KRT5 and SERPINB4 immunofluorescence in control mucosa, non-eosinophilic CRSwNP, and eosinophilic CRSwNP tissues. The representative fields showed altered epithelial marker organization and SERPINB4-associated staining in CRSwNP tissues, particularly in eosinophilic disease (Figure 3G). These images are presented as qualitative visual support rather than quantitative validation.

### 3.4. Independent Validation Provides Partial and Uneven External Support for Framework-Related Features

**Figure 4:**
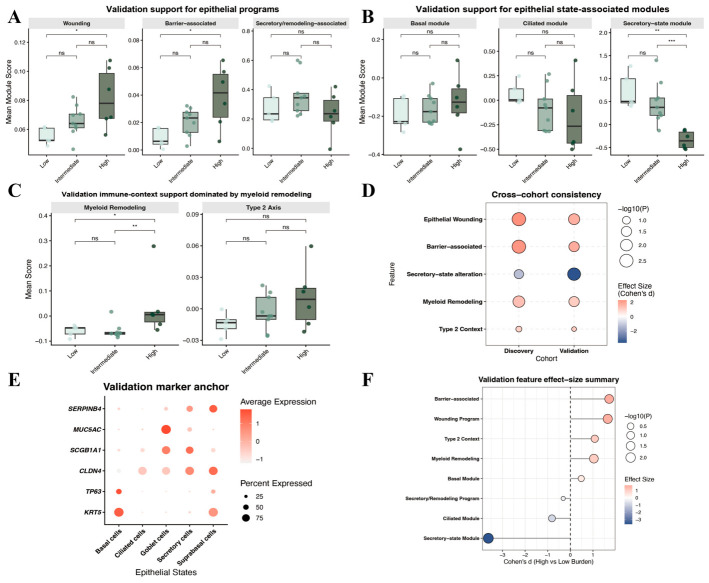
Independent scRNA-seq validation provides partial and uneven external support for framework-related features. (**A**) Validation support for epithelial programs. Wounding and barrier-associated programs showed the clearest directional changes across burden-oriented comparison groups, whereas secretory/remodeling-associated changes were less uniform. (**B**) Validation support for epithelial state-associated modules. Module-level shifts were observed in selected epithelial state programs, but these changes were not uniform across all state-associated features. (**C**) Immune-context support. Myeloid remodeling showed clearer support than the type 2 T/NK context axis, which was weaker and uneven. (**D**) Cross-cohort effect-size comparison of selected discovery and validation features. Effect sizes were derived from compartment-matched sample-level summaries and are intended to summarize directional compatibility rather than exact replication of the discovery composite. (**E**) Validation marker anchor for selected epithelial features. (**F**) Validation feature effect-size summary, highlighting stronger support for wounding-, barrier-, and myeloid-remodeling-related features and weaker or more variable support for other axes. Statistical significance is indicated as ns, not significant; * *p* < 0.05; ** *p* < 0.01; *** *p* < 0.001.

The independent scRNA-seq validation cohort provided partial and uneven external support for the framework-related features. Because this dataset lacked explicit eosinophilic subclassification and did not allow exact reconstruction of all four discovery axes, we evaluated conceptually aligned epithelial and immune modules in their corresponding compartments. At the epithelial program level, wounding and barrier-associated programs showed the clearest directional changes across the burden-oriented comparison groups, whereas the secretory/remodeling-associated program was less uniform (Figure 4A). At the epithelial state level, burden-driven differences were more pronounced in transcriptional modules than in direct state abundance. Among the modules examined, the strongest signal emerged along the secretory axis, with module scores progressively decreasing toward the higher-burden comparison groups. Ciliated cell features exhibited a weaker downward trend, while the basal module remained relatively stable (Figure 4B). In the immune compartment, the myeloid remodeling panel showed clearer support than the type 2 T/NK context panel, whose comparisons were weak or non-significant (Figure 4C). Cross-cohort effect-size comparisons revealed that shared signals were strongest for epithelial wounding and barrier programs, alongside myeloid remodeling; in contrast, secretory state alterations and type 2 context were weaker or more variable in the external cohort (Figure 4D). A compact epithelial marker panel provided additional gene-level anchoring for basal, barrier, canonical secretory, and pathology features (Figure 4E). Within the validation cohort itself, feature-level effect-size decomposition again highlighted the wounding, barrier, and myeloid remodeling axes as the most robust, while other epithelial and immune features remained variable (Figure 4F). Supporting validation metrics—including epithelial atlas structure, score projections, relative abundance summaries, and sample-level feature correlations—are provided in Figure S4A–E. Thus, the validation data support selected epithelial injury, barrier-associated, and myeloid remodeling features, but they do not demonstrate uniform replication across all framework-related axes.

### 3.5. GeoMx Spatial Profiling Provides Compartment-Resolved Support While Illustrating Heterogeneous Molecular Organization Across Nominal Disease Groups

**Figure 5:**
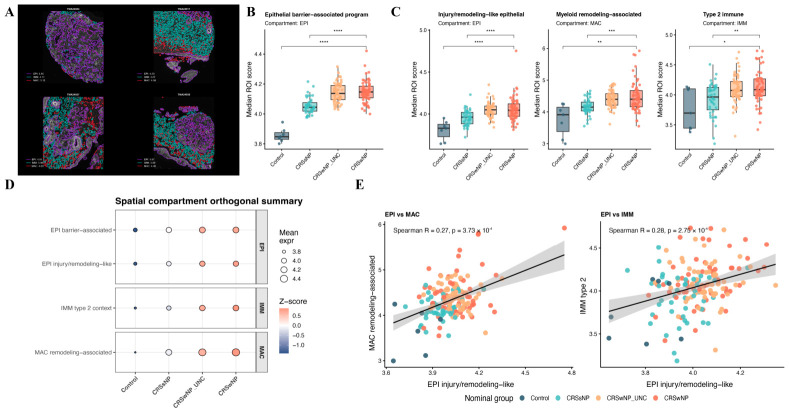
GeoMx spatial profiling provides compartment-resolved support while illustrating heterogeneous molecular organization across nominal disease groups. (**A**) Representative FOVs from four nominal disease groups showing compartment-level molecular support in GeoMx spatial profiling. For each FOV, compartment outlines are displayed together with compartment-specific scores using an epithelial injury/remodeling-like program in EPI, an immune type 2-context score in IMM, and a macrophage remodeling-associated score in MAC. These representative FOVs are shown to illustrate compartment-level heterogeneity across nominal groups and are not intended to represent a monotonic progression series. (**B**) A consistent epithelial barrier-associated axis across nominal disease groups in the epithelial compartment. Each dot represents one ROI-level summary value; boxplots show median and interquartile range. (**C**) Compartment-resolved burden-associated features across the GeoMx cohort, including an epithelial injury/remodeling-like program in EPI, a macrophage remodeling-associated profile in MAC, and an immune type 2-context score in IMM. Each dot represents one ROI-level summary value; boxplots show median and interquartile range. (**D**) Relative cross-compartment summary of key spatial features across nominal disease groups. Dot color indicates relative z-scored mean expression/score within each compartment-feature axis, and dot size indicates mean signal magnitude. This panel is intended as a descriptive summary of compartment-level organization. (**E**) ROI-level cross-compartment relationships in GeoMx spatial profiling. The left panel shows the relationship between epithelial injury/remodeling-like and macrophage remodeling-associated features, and the right panel shows the relationship between epithelial injury/remodeling-like and immune type 2-context features. Each dot represents one ROI; colors indicate the nominal disease group. The observed correlations were modest and are interpreted as partial cross-compartment coupling rather than strong predictive relationships. Substantial overlap and heterogeneity across disease labels remained. Statistical significance is indicated as * *p* < 0.05; ** *p* < 0.01; *** *p* < 0.001; **** *p* < 0.0001.

To extend the burden framework into a spatial dataset, we analyzed a public GeoMx cohort at the compartment level, focusing on four core modules: epithelial injury/remodeling, epithelial barrier, macrophage remodeling, and type 2 immune context. Representative FOVs across nominal disease groups showed marked compartment-level heterogeneity rather than an ordered progression pattern, indicating that spatial molecular states did not map one-to-one onto nominal labels (Figure 5A). At the cohort level, the epithelial barrier program was consistently elevated relative to controls in the epithelial compartment (Figure 5B). Compartment-resolved analyses further showed that the clearest spatial signals arose from the epithelial injury/remodeling and macrophage remodeling profiles, whereas the type 2 immune context was directionally consistent but more modest (Figure 5C). A relative cross-compartment summary highlighted coordinated enrichment of epithelial injury, barrier, and macrophage remodeling signals across nominal disease groups, while the type 2 immune axis provided additional contextual information (Figure 5D). At the ROI level, epithelial injury/remodeling features correlated positively with both macrophage remodeling and type 2 immune context features, yet substantial overlap across nominal groups remained evident (Figure 5E). Compartment identity verification, an immune/myeloid area-fraction proxy, and a detailed ROI-level feature matrix are shown in Figure S5A–C. These spatial findings are compatible with compartment-resolved molecular organization related to the burden framework, while also showing substantial ROI-level heterogeneity across nominal disease groups.

### 3.6. Independent Bulk Transcriptomic Cohorts Support the Burden Framework and Further Illustrate Incomplete Concordance Between Nominal Eosinophilic Labels and Molecular Burden

**Figure 6:**
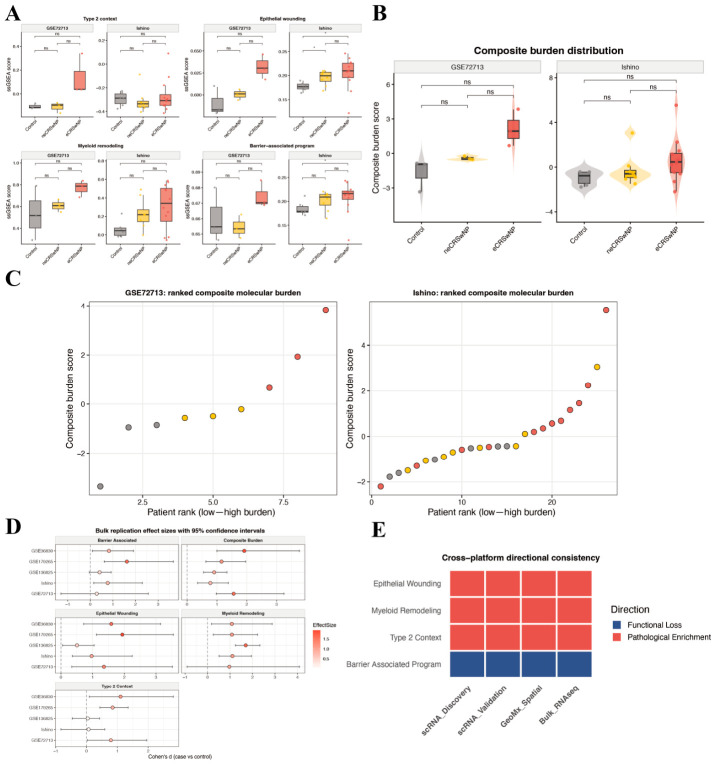
Independent bulk transcriptomic cohorts support the burden framework and incomplete concordance between nominal eosinophilic labels and molecular burden in CRSwNP. (**A**) Directional support for representative burden-associated component features in two label-explicit bulk cohorts, GSE72713 and Ishino. Scores for type 2 context, epithelial wounding, myeloid remodeling, and barrier-associated programs are shown across control, non-eosinophilic CRSwNP, and eosinophilic CRSwNP samples. These data are interpreted primarily in terms of directional compatibility and relative effect magnitude rather than strict categorical separation. (**B**) Composite molecular burden distributions in GSE72713 and Ishino. In both cohorts, eosinophilic CRSwNP samples tend to occupy the higher-burden range, whereas non-eosinophilic CRSwNP samples show partial overlap with both controls and eosinophilic CRSwNP. (**C**) Ranked sample-level composite molecular burden in GSE72713 and Ishino. Samples are ordered from low to high burden within each cohort to illustrate graded molecular organization and incomplete concordance across nominal eosinophilic labels. (**D**) Multi-cohort bulk replication effect sizes with 95% confidence intervals. Points show Cohen’s d for case-versus-control comparisons within each cohort-feature axis, and horizontal intervals show bootstrap 95% confidence intervals. Positive values indicate higher scores in CRSwNP or nasal polyp samples relative to controls. Composite burden, epithelial wounding, and myeloid remodeling showed more consistent positive effects across cohorts than type 2 context, although confidence interval width and statistical support varied by cohort and feature. (**E**) Cross-platform directional summary of key burden-associated features across discovery scRNA-seq, validation scRNA-seq, GeoMx spatial profiling, and bulk transcriptomic analyses. Bulk RNA-seq summarizes the overall directional pattern observed across the bulk validation layer rather than a single individual cohort. Statistical significance is indicated as ns, not significant; * *p* < 0.05.

Finally, we examined whether the burden framework could be reproduced in independent bulk transcriptomic cohorts. We first focused on two explicitly annotated bulk datasets, GSE72713 and Ishino, which categorized samples into control, non-eosinophilic CRSwNP, and eosinophilic CRSwNP groups. Across both cohorts, the core burden features exhibited overall directional consistency with increasing disease burden, although the strength of statistical separation varied by cohort and individual feature. Among the four axes, epithelial wounding showed the clearest and most reproducible increase. Myeloid remodeling and barrier features followed similar trends but were more variable, while the type 2 immune context yielded weaker signals in the bulk setting (Figure 6A). Composite burden analysis revealed that eosinophilic CRSwNP samples predominantly occupied the high-burden range, whereas controls clustered at the low-burden end, and non-eosinophilic cases spanned the intermediate space. However, overlap across nominal groups remained evident in both datasets, confirming that pathology-based eosinophilic labels are informative but do not map one-to-one onto the underlying molecular burden landscape (Figure 6B).

This supports incomplete concordance between nominal eosinophilic labels and bulk molecular burden, but the strength of separation varied across features and cohorts.

Ranking individual samples within each cohort further illustrated this continuous, graded organization. In both GSE72713 and Ishino, eosinophilic CRSwNP samples were enriched at the high-burden pole, yet non-eosinophilic CRSwNP samples were clearly not restricted to the low-burden range, leaving distinct overlap across traditional clinical boundaries (Figure 6C).

The updated multi-cohort effect-size summary now reports Cohen’s d with 95% confidence intervals (Figure 6D). Composite burden was positive in all five bulk cohorts (d = 0.779–1.916), although statistical support and interval width varied. Epithelial wounding was also positive across cohorts (d = 0.508–1.936), with stronger support in several cohorts. Myeloid remodeling was consistently positive (d = 0.969–1.700), but some confidence intervals were wide, especially in small label-explicit cohorts. In contrast, the type 2 context was less consistent, with near-null effects in Ishino and GSE136825 and stronger effects only in selected cohorts, with confidence intervals indicating substantial between-cohort variability. These results support the composite, epithelial wounding, and myeloid remodeling features more consistently than the type 2 context alone.

The cross-platform directional summary is shown in Figure 6E. Additional bulk summaries include raw CT-versus-NP composite distributions, patient-level feature landscapes in Ishino and GSE72713, and LOCO internal-consistency checks (Figure S6A–D). Alternative-signature sensitivity analyses further supported the main directional bulk interpretation (Figure S6E). Replacing the curated type 2 panel with a public type 2 immune response signature and expanding the myeloid remodeling panel preserved positive alternative composite effect sizes across cohorts. Original and alternative myeloid remodeling scores were strongly correlated across cohorts (Spearman r = 0.901–0.967), whereas type 2 correlations were more variable (Spearman r = 0.032–0.600), consistent with greater sensitivity of type 2 scoring to gene-set definition and platform context. Original and alternative composite scores remained moderately to strongly correlated (Spearman r = 0.639–0.950), and high-burden tertile overlap ranged from 0.667 to 0.889. These sensitivity analyses indicate that the bulk conclusions are not restricted to one curated signature definition, while also showing that individual axes, especially type 2 context, should be interpreted cautiously.

## 4. Discussion

Our integrative multimodal analyses suggest that nominal eosinophilic labels in CRSwNP, while clinically informative, do not fully capture the molecular complexity of diseased tissue. Across single-cell, spatial transcriptomic [24], and bulk layers, the most reproducible disease-burden signals centered on epithelial injury and myeloid remodeling axes. This pattern aligns with growing evidence that CRSwNP tissue biology is shaped not solely by conventional inflammation, but by epithelial dysfunction, remodeling, and complex immune–epithelial interactions [14,16,25,26,27].

While eosinophilic subclassification remains clinically meaningful [1,2,9], our analyses suggest that it captures only a fraction of the underlying tissue-state heterogeneity. In the discovery cohort, histologic eosinophilic labels correlated with molecular burden, but this relationship was incomplete: nominally non-eosinophilic CRSwNP samples were not restricted to the low-burden end, frequently extending into the intermediate-to-high range. This discordance was also observed across independent label-explicit bulk cohorts (GSE72713 and Ishino). In both datasets, eosinophilic samples tended to occupy the higher-burden spectrum, whereas non-eosinophilic cases were distributed more broadly, resulting in substantial overlap across traditional clinical boundaries. These findings support an exploratory, graded model of CRSwNP molecular organization rather than a strictly categorical one.

This molecular continuum is biologically plausible in light of the current classification landscape. While eosinophilic subclassification retains clinical utility, its operational definitions vary widely across studies, regions, and practice settings [9,10,11]. For instance, EPOS 2020 considers tissue eosinophilia to be one indicator supporting type 2 inflammatory disease, not a universal, standalone histologic standard [1]. Conversely, JESREC utilizes a composite framework incorporating clinical, radiologic, and blood eosinophil metrics, avoiding reliance on a single tissue threshold [10]. Similarly, updated Chinese guidelines emphasize the absence of unified diagnostic criteria, acknowledging diverse metrics such as eosinophil proportions, counts per high-power field, and recurrence-based thresholds [9,11]. Geographic variations in CRS inflammatory signatures—particularly regarding type 2 prevalence and eosinophilic predominance—add another layer of complexity [17]. Against this backdrop, the incomplete correspondence between nominal eosinophilic labels and molecular burden is compatible with underlying biological heterogeneity.

A major observation of this study is that epithelial injury and remodeling features provided the most stable disease-burden signals across analytical platforms. In the discovery single-cell dataset, higher burden was reflected mainly by epithelial state reorganization, amplified wounding programs, and barrier alterations. In the independent validation cohort, the largest shifts again centered on the wounding and barrier axes, whereas epithelial state and immune features were less uniform. The bulk datasets showed a similar pattern: composite burden exhibited the strongest and most consistent case-versus-control effect, while epithelial wounding and myeloid remodeling also demonstrated broadly concordant positive effects. By comparison, the type 2 context was weaker, and barrier features, although directionally consistent, varied more across datasets. These observations align with the emerging consensus that CRSwNP is shaped not solely by immune inflammation, but by epithelial dysfunction, altered differentiation, impaired mucosal defense, and chronic remodeling [14,16,25]. Indeed, tight-junction disruption, barrier breakdown, and defective host defense have been repeatedly implicated in CRS pathophysiology [14,16] and likely perpetuate persistent tissue remodeling [28]. Recent single-cell and spatial atlases similarly highlight epithelial programs and immune–epithelial crosstalk, particularly those involving basal progenitor trajectories and remodeling-linked states, as central architectural features of nasal polyp biology [16,18]. Our findings extend this literature by suggesting that epithelial injury and remodeling dimensions are among the more reproducible axes spanning nominal eosinophilic categories. The immunofluorescence panel provided qualitative in situ support for epithelial remodeling features, not quantitative validation.

Notably, individual modules did not perform uniformly across datasets. Type 2 inflammation remains clinically important and therapeutically actionable in severe CRSwNP, as evidenced by current guidelines and the efficacy of type 2-targeted biologics [1,5,29,30]. However, in our bulk replication cohorts, the type 2 signature was less consistent than composite burden, epithelial wounding, or myeloid remodeling. Similarly, barrier and host-defense features displayed substantial cross-cohort variance. This variability does not diminish the clinical relevance of type 2 inflammation; it indicates that tissue-level transcriptomic organization cannot be reduced to a single inflammatory axis. Different analytical platforms and cohorts may capture immune signals disparately, particularly within biologically heterogeneous CRSwNP populations [17,18,25]. Furthermore, because this study relies on retrospective datasets spanning diverse sampling schemes, compartments, and assay structures, these cross-platform differences warrant cautious interpretation. Technical covariates, including tissue-site heterogeneity, cellular averaging in bulk RNA-seq, and within-individual dependence in single-cell analyses, can affect apparent effect sizes across datasets [31].

Among non-epithelial components, the most consistent signals arose from myeloid remodeling features rather than a uniformly strong type 2 context. In the validation single-cell dataset, myeloid remodeling provided the clearest non-epithelial signal, whereas the type 2 T/NK axis offered weaker directional support. Similarly, in the spatial dataset, the strongest compartment-level support emerged from epithelial injury and macrophage remodeling profiles, while type 2 immune scores were directionally concordant but more modest. The external layers, therefore, provide directional evidence for selected framework features, without exact one-to-one replication; in particular, the validation single-cell and GeoMx datasets lacked a stromal/fibroblast compartment comparable to the discovery extracellular-matrix axis. This pattern fits mechanistic frameworks linking macrophage remodeling, coagulation cascade imbalances, and extracellular matrix organization to nasal polyp biology, including evidence for excessive fibrin deposition and impaired fibrinolysis [28]. Recent cellular studies also connect specific myeloid states with type 2 pathophysiology and immune recruitment in eosinophilic CRSwNP [18]. Consequently, epithelial and myeloid remodeling states appear to be relatively stable organizational dimensions within our burden framework, even when immune signals vary across platforms [16,18]. This tissue-state view is consistent with recent interpretations that eosinophil- and neutrophil-associated inflammation in CRS should be considered within a broader tissue context rather than treated as mutually exclusive categories [32]. Histopathologic descriptions of CRSwNP nasal mucosa, including stromal edema, basement membrane thickening, and eosinophil-predominant inflammatory infiltrates, further fit this interpretation [33]. Eosinophilic subclassification, therefore, remains clinically relevant, but pathology-based labels and multidimensional molecular features appear to capture partially overlapping aspects of CRSwNP tissue biology. Prospective cohorts with harmonized eosinophilic definitions, matched single-cell/spatial/bulk profiling, and quantitative tissue validation will be needed before prognostic or therapeutic utility is inferred.

## 5. Conclusions

In conclusion, our integrative analysis of public single-cell, spatial, and bulk transcriptomic datasets suggests that nominal eosinophilic labels in CRSwNP, while clinically informative, do not fully capture the underlying molecular complexity of diseased tissue. Across analytical platforms, epithelial injury/remodeling, barrier-associated disruption, and remodeling-linked myeloid features were more consistently observed than a uniformly strong type 2 signal. These findings support an exploratory graded model of CRSwNP molecular organization that may complement conventional pathology-based eosinophilic subclassification, although prospective harmonized cohorts will be required before clinical use.

## Data Availability

The public datasets analyzed in this study were obtained from the original published studies and public repositories cited in the Methods and References. In addition, original immunofluorescence images generated for this study are available from the corresponding author upon reasonable request.

## Supplementary Materials

The following supporting information can be downloaded at: https://www.mdpi.com/article/10.3390/biomedicines14061189/s1. Figure S1: Supplementary support for burden construction and discovery-cohort burden organization; Figure S2: Additional support for burden stratification and robustness in the discovery cohort; Figure S3: Supplementary support for epithelial-focused single-cell analysis; Figure S4: Supplementary single-cell support for the external validation epithelial cohort; Figure S5: Supplementary spatial support for compartment-resolved GeoMx analyses; Figure S6: Supplementary support for independent bulk-cohort replication of the burden framework; Table S1: Discovery molecular burden signatures and retained gene counts after filtering to the pseudobulk discovery scRNA-seq matrix.

## Abbreviations

The following abbreviations are used in this manuscript:

CRS	chronic rhinosinusitis
CRSwNP	chronic rhinosinusitis with nasal polyps
CRSsNP	chronic rhinosinusitis without nasal polyps
HRQoL	health-related quality of life
JESREC	Japanese Epidemiological Survey of Refractory Eosinophilic Chronic Rhinosinusitis
scRNA-seq	single-cell RNA sequencing
GeoMx	GeoMx digital spatial profiling
ECM	extracellular matrix
TMM	trimmed mean of M values
logCPM	log counts per million
GSVA	gene set variation analysis
ssGSEA	single-sample gene set enrichment analysis
t-SNE	t-distributed stochastic neighbor embedding
ROI	region of interest
FOV	field of view
HPF	high power field
ECRS	eosinophilic chronic rhinosinusitis
T/NK	T cell/natural killer cell
CT	control tissue
NP	nasal polyp
