# Supplementary material for: Incomplete Concordance Between Nominal Eosinophilic Labels and Molecular Burden in Chronic Rhinosinusitis with Nasal Polyps
Source: Biomedicines. 2026 May 25;14(6):1189. doi: 10.3390/biomedicines14061189 (PMC13296064; doi:10.3390/biomedicines14061189)
Supplement: Supplementary file 1 [file biomedicines-14-01189-s001.zip › biomedicines-4283698-supplementary.pdf]

Supplementary Figures and Table

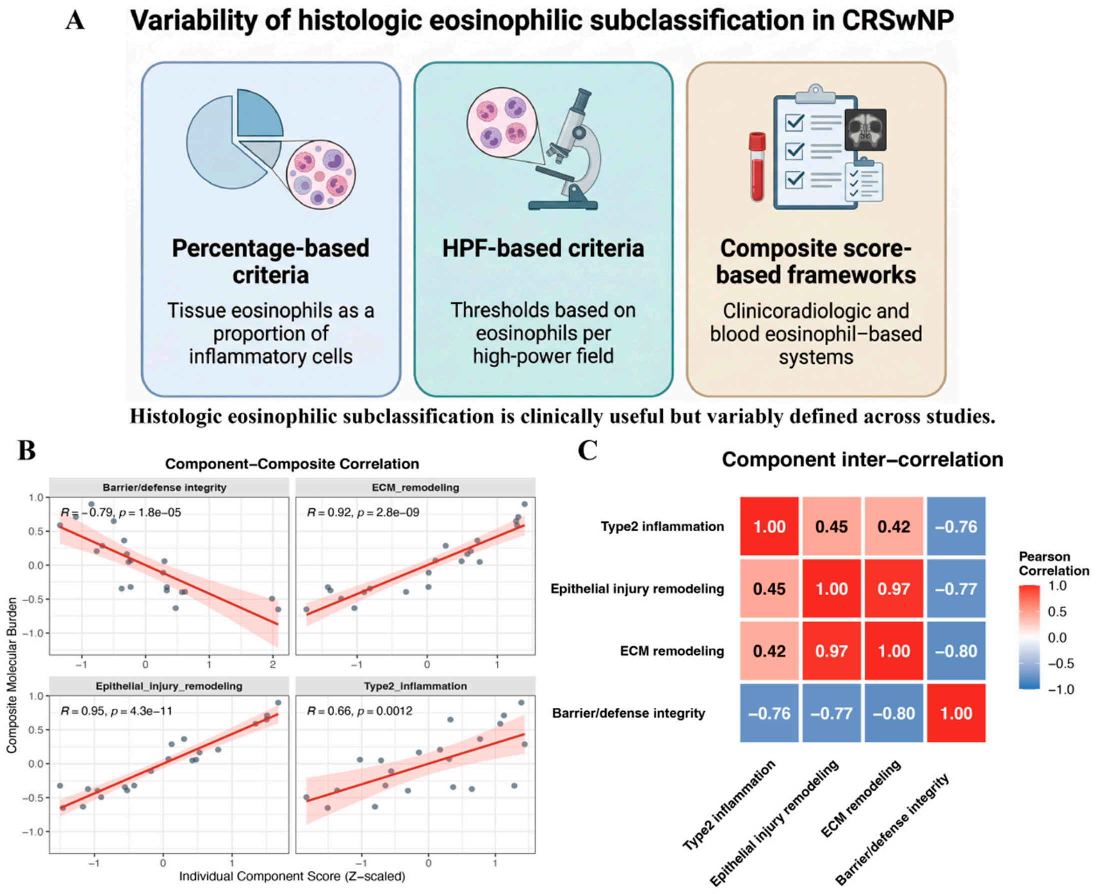

Figure S1. Supplementary support for burden construction and discovery-cohort burden organization.

(A) Variability of histologic eosinophilic subclassification in CRSwNP. Schematic summary of commonly used approaches, including percentage-based criteria, eosinophils-per-high-power-field thresholds, and composite score-based frameworks.

(B) Correlation of individual burden components with the composite molecular burden score in the discovery cohort. Epithelial injury/remodeling and extracellular-matrix remodeling show the strongest positive correlations, whereas barrier/defense integrity shows an opposite directional relationship.

(C) Inter-correlation matrix of the four burden components. Type 2 inflammation, epithelial injury/remodeling, and extracellular-matrix remodeling align in the same overall direction, whereas barrier/defense integrity shows the opposite trend consistent with its protective role.

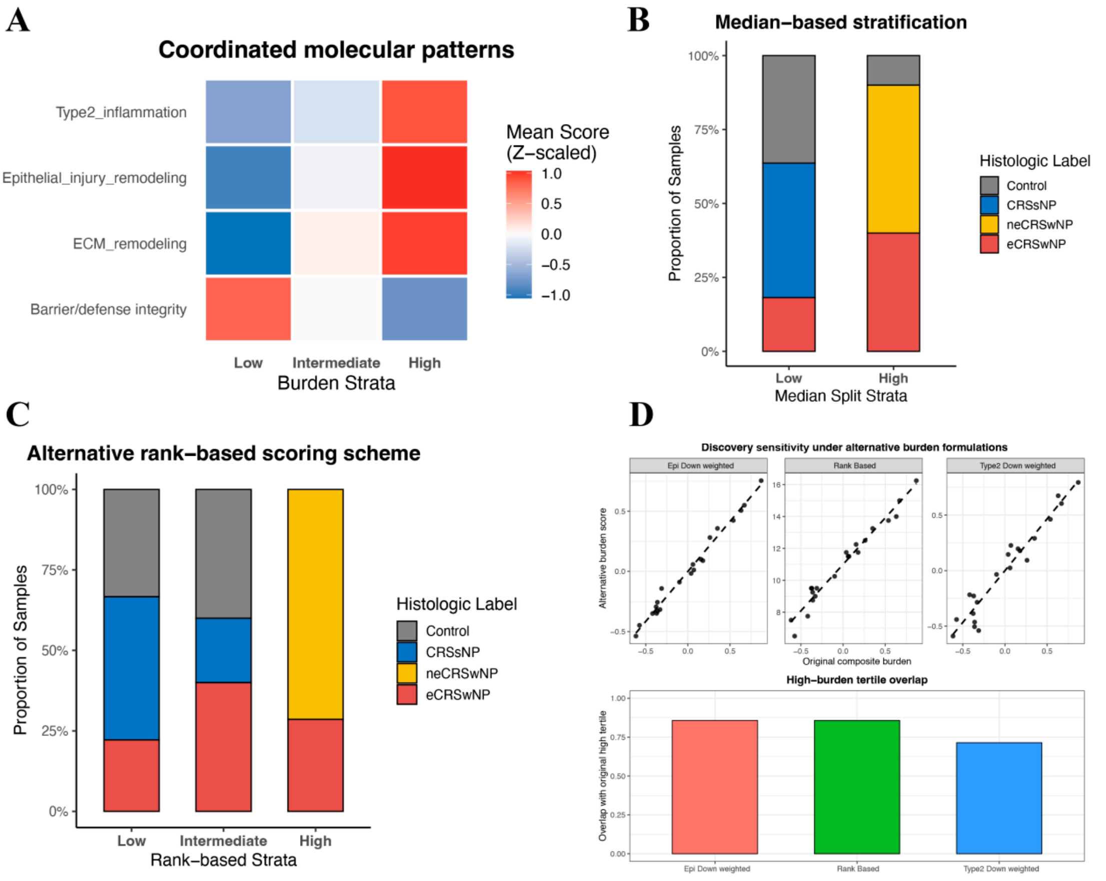

Figure S2. Additional support for burden stratification and robustness in the discovery cohort.

(A) Heatmap-based summary of the mean burden component scores across low-, intermediate-, and high-burden strata. This alternative visualization is consistent with the component-wise quantitative comparison shown in Fig. 2E and shows a contrasting directional pattern for the barrier/defense integrity component relative to the burden-associated dimensions.

(B) Distribution of histologic labels after reclassifying samples into low- and high-burden groups using the cohort median of the composite molecular burden score. The overall burden-associated pattern remained directionally consistent with the tertile-based stratification shown in Fig. 2B.

(C) Distribution of histologic labels under an alternative rank-based molecular burden scoring scheme. The overall burden-associated label pattern remained directionally consistent, providing an additional descriptive check of the sample-level burden representation.

(D) Discovery sensitivity under alternative burden formulations. Alternative discovery composites were generated by downweighting epithelial injury/remodeling, downweighting type 2 inflammation, or using a rank-based composite. Scatterplots compare each alternative score with the original equal-weighted composite, and the bar plot shows overlap with the original high-burden tertile. These analyses assess sensitivity to reasonable alternative weighting choices.

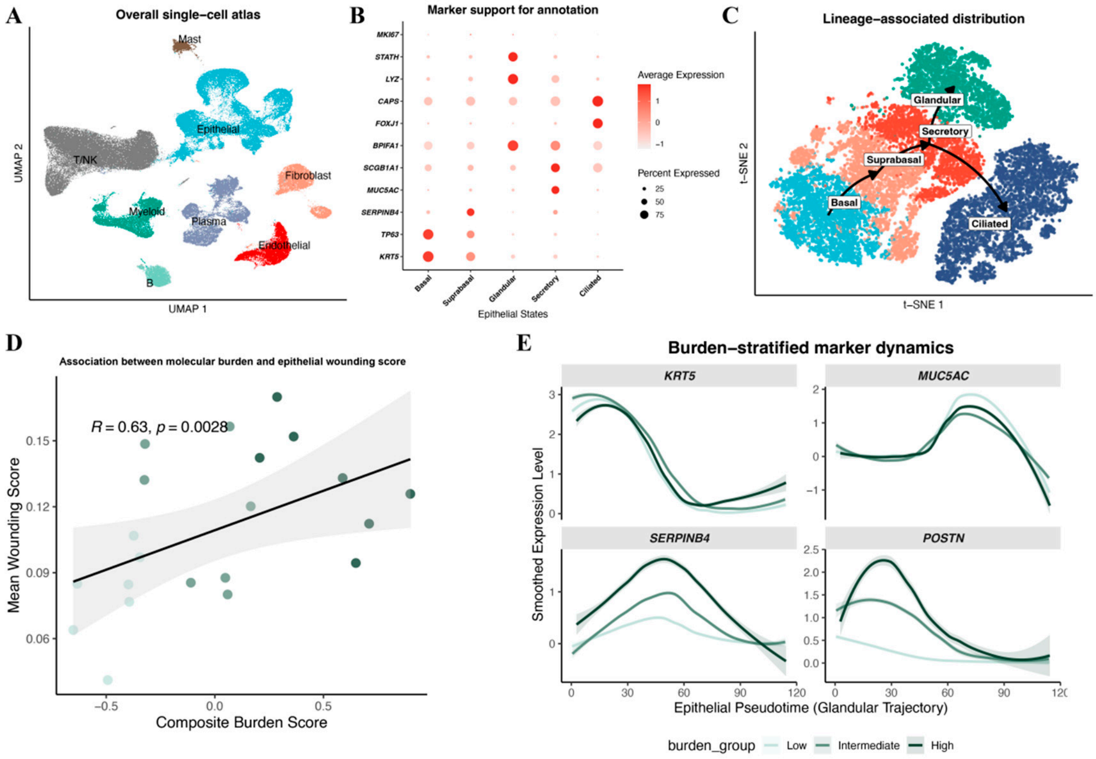

Figure S3. Supplementary support for epithelial-focused single-cell analysis.

(A) Overall single-cell atlas of the discovery cohort with major cell-type annotations, showing the epithelial compartment from which the focused epithelial analysis in Fig. 3 was derived.

(B) Marker support for epithelial substate annotation. Broader marker expression patterns were used to support the annotation of epithelial substates shown in Fig. 3A.

(C) Lineage-associated distribution of epithelial substates in low-dimensional space. Slingshot-based lineage structure provided supplementary trajectory support for the epithelial pseudotime analyses shown in Fig. 3E–F.

(D) Continuous association between molecular burden and epithelial wounding score. Sample-level burden remained positively associated with epithelial injury-related program activity in the epithelial compartment.

(E) Burden-stratified marker dynamics along the glandular epithelial trajectory. Smoothed expression curves of representative genes provided additional support for burden-dependent differences in branch-level epithelial dynamics.

—

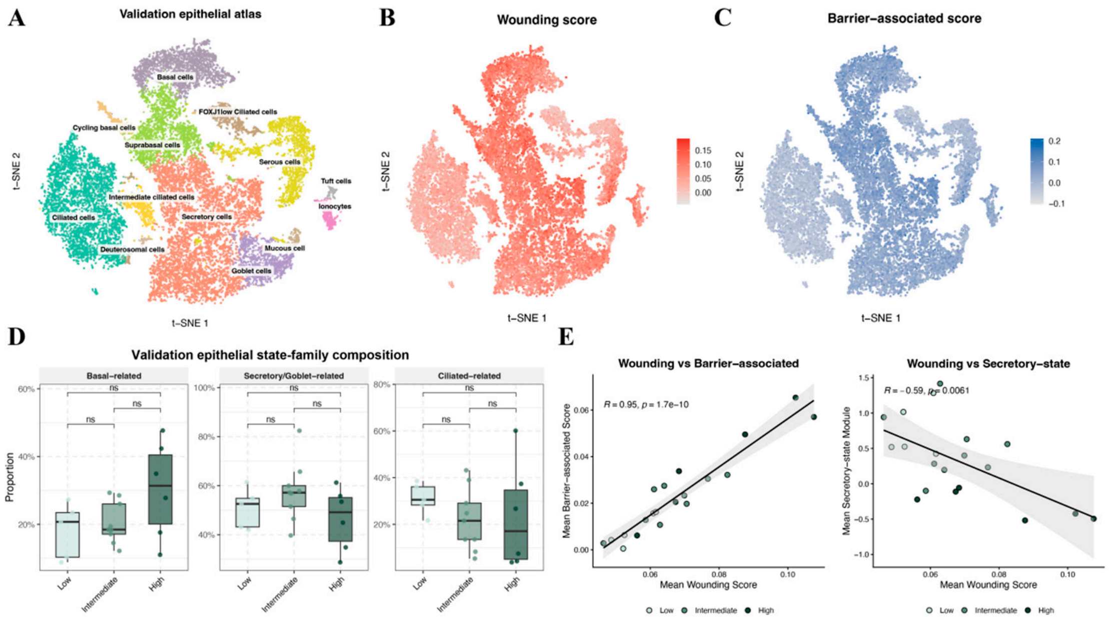

Figure S4. Supplementary single-cell support for the external validation epithelial cohort.

(A) Labeled t-SNE projection of the validation epithelial atlas, providing the structural reference for downstream module-level interpretation.

(B) Wounding score projection across the validation epithelial atlas. Wounding-associated activity was not uniformly distributed across epithelial cells and showed enrichment in specific epithelial regions and states.

(C) Barrier-associated score projection across the validation epithelial atlas. Similar to the wounding program, barrier-associated activity showed a non-random distribution across epithelial states.

(D) Validation epithelial state-family composition across burden-oriented comparison groups, summarized for basal-related, secretory/goblet-related, and ciliated-related epithelial families.

(E) Sample-level epithelial feature correlation summary, showing the positive coupling between wounding and barrier-associated programs and the inverse relationship between wounding and the canonical secretory-state module.

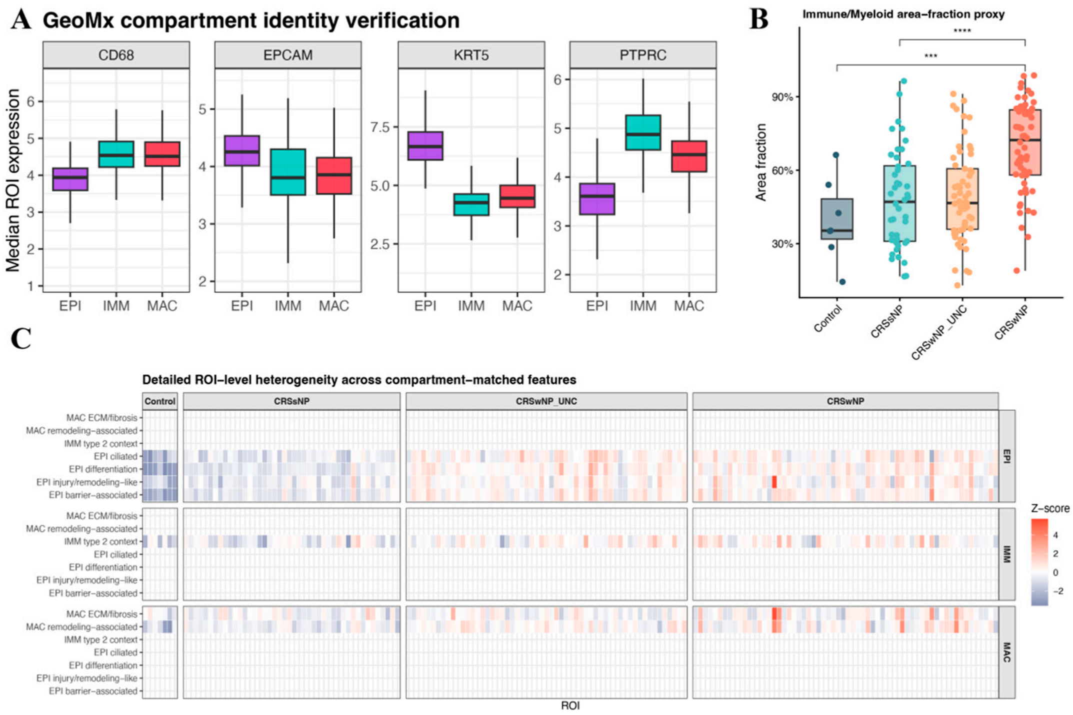

Figure S5. Supplementary spatial support for compartment-resolved GeoMx analyses.

(A) Compartment identity verification for the GeoMx segmentation strategy. Established lineage markers are shown across EPI, IMM, and MAC compartments to support compartment assignment.

(B) Immune/myeloid area-fraction proxy across nominal disease groups. Each dot represents one ROI-level value; boxplots show median and interquartile range. This panel provides supportive spatial-context information and is not intended as a primary burden-defining axis.

(C) Detailed ROI-level heterogeneity across compartment-matched features. Values are z-scored within each feature to visualize relative heterogeneity across ROIs and nominal disease groups.

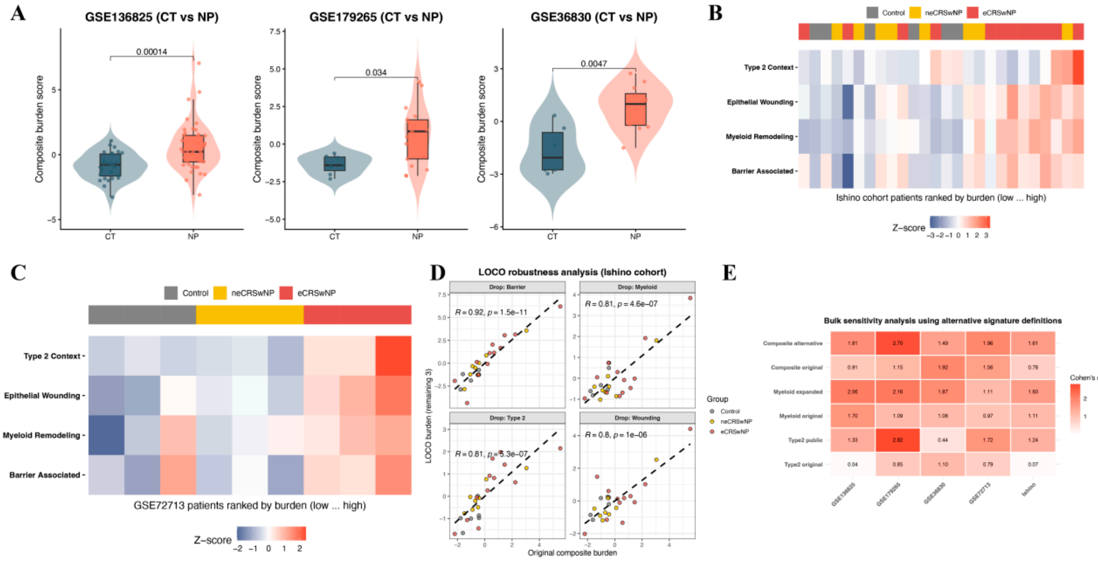

Figure S6. Supplementary support for independent bulk-cohort replication of the burden framework.

(A) Raw composite burden distributions across supporting independent CT-versus-NP bulk cohorts. These plots provide distribution-level context for the summarized cross-cohort comparisons shown in Fig. 6D and illustrate that replication strength varies across cohorts.

(B) Patient-level burden-feature landscape in the Ishino cohort. Samples are ordered from low to high composite burden, with the annotation bar indicating nominal group labels and the heatmap showing z-scored values of the major burden-associated component features. This panel highlights graded and heterogeneous molecular organization across individual samples.

(C) Patient-level burden-feature landscape in GSE72713. Samples are ordered from low to high composite burden, with the annotation bar indicating nominal group labels and the heatmap showing z-scored values of the major burden-associated component features. This panel provides an additional label-explicit view of heterogeneous burden organization across individual samples.

(D) Concordance between the original composite burden score and leave-one-component-out scores is shown as an internal consistency check.

(E) Bulk sensitivity analysis using alternative signature definitions. Type 2 context was rescored using a public type 2 immune response gene set, and myeloid remodeling was rescored using an expanded myeloid/remodeling panel. The heatmap summarizes original and alternative effect sizes across bulk cohorts. These analyses support the main directional bulk conclusions while showing that type 2 estimates are more sensitive to signature definition.

Supplementary Table S1. Discovery molecular burden signatures and retained gene counts after filtering to the pseudobulk discovery scRNA-seq matrix.

Framework feature	MSigDB source/signature	Genes in source set	Genes retained in discovery matrix
Type 2 inflammatory context	GOBP_TYPE_2_IMMUNE_RESPONSE	37	35
Epithelial injury/remodeling	HALLMARK_EPITHELIAL_MESENCHYMAL_TRANSITION	200	200
Extracellular-matrix remodeling	REACTOME_EXTRACELLULAR_MATRIX_ORGANIZATION	301	297
Barrier integrity	HALLMARK_APICAL_JUNCTION; HALLMARK_APICAL_SURFACE	239	237
Antibacterial defense	GOBP_DEFENSE_RESPONSE_TO_BACTERIUM	361	296

Note: Retained genes indicate genes from each source signature that were present in the discovery pseudobulk expression matrix used for GSVA/ssGSEA scoring.
